# Optimization Conditions for High-Power AlGaN/InGaN/GaN/AlGaN High-Electron-Mobility Transistor Grown on SiC Substrate

**DOI:** 10.3390/ma17225515

**Published:** 2024-11-12

**Authors:** Bonghwan Kim, Seung-Hwan Park

**Affiliations:** Department of Semiconductor Electronic Engineering, Daegu Catholic University, Gyeongsan 38430, Gyeongbuk, Republic of Korea; shpark@cu.ac.kr

**Keywords:** AlGaN/InGaN/GaN/AlGaN/SiC, HEMT, TCAD

## Abstract

In this study, we aimed to propose an optimal structure for an AlGaN/InGaN/GaN/AlGaN/SiC HEMT by investigating how the breakdown voltage varies with the thickness and composition of the InGaN layer. The breakdown voltage was shown to be highly dependent on the In composition. Specifically, as the In composition increased, the breakdown voltage rapidly increased, but it exhibited saturation when the In composition exceeded 0.06. Therefore, it is desirable to maintain the In composition at or above 0.06. The variation in breakdown voltage due to thickness was relatively small compared to the variation caused by In composition. While the breakdown voltage remained nearly constant with increasing thickness, it began to decrease when the thickness exceeded 10 nm. Hence, the thickness should be kept below 10 nm. Additionally, as the In composition increased, the subthreshold swing (SS) also increased, but the drain current value was shown to increase. On the other hand, it was observed that the SS value in the transfer characteristics and the current–voltage characteristics were almost unaffected by the thickness of the InGaN layer.

## 1. Introduction

Gallium nitride (GaN)-based high-electron-mobility transistors (HEMTs) have garnered significant attention due to their applications in power electronics, high-frequency communication systems, and RF amplifiers [1,2,3,4,5].

One of the critical parameters of HEMTs is the breakdown voltage, which is closely linked to device reliability. A higher breakdown voltage ensures better resilience to sudden voltage variations and spikes, increasing the device’s lifetime and preventing damage caused by overvoltage. Consequently, high-breakdown-voltage HEMTs can operate stably under a high voltage, making them suitable for power conversion and high-voltage electronic systems. Therefore, developing HEMTs with a high breakdown voltage is essential for enhanced reliability and broader application in high-voltage environments.

Several approaches to increasing the breakdown voltage of GaN-based HEMTs have been proposed, with one common method being the introduction of a back-barrier layer [6,7,8,9,10]. It has been demonstrated that replacing the GaN channel in conventional HEMTs with an InGaN/GaN coupling channel leads to a higher breakdown voltage than traditional GaN channel HEMTs [6]. However, the optimization of such structures remains largely unexplored. In this work, we aim to propose an optimized structure by investigating how the thickness and composition of the InGaN layer in AlGaN/InGaN/GaN/AlGaN/SiC HEMTs affect the breakdown voltage. The characteristics of the device were simulated using the Silvaco technology computer-aided design (TCAD) simulation tool [11].

## 2. Methods

To investigate the effect of the thickness and composition of the InGaN layer on the breakdown voltage in AlGaN/InGaN/GaN/AlGaN/SiC HEMTs, similar to the structure studied by Murugapandiyan et al. [4], simulations were conducted using the Silvaco TCAD tool (version 5.2.23.R).

The device structure was modeled as follows:

- A silicon carbide (4H-SiC) substrate was used as the base material due to its high thermal conductivity and ability to handle high power.
- A 1 µm thick Al_0.05_Ga_0.95_N back-barrier layer was deposited on top of the substrate.
- A 10 nm thick GaN layer was used to form the primary channel for electron transport.
- The InGaN layer, whose thickness and composition were varied in the simulation, was placed on top of the GaN layer.
- A 10 nm thick Al_0.3_Ga_0.7_N barrier layer was used to control electron confinement in the channel region.
- A 500 nm thick silicon nitride (SiN) passivation layer was deposited to reduce surface states and prevent surface-related traps.

In the simulation, the InGaN layer’s composition was varied between 0.02 and 0.10 to observe the effect of indium content on the breakdown voltage. Additionally, the thickness of the InGaN layer was varied between 5 nm and 20 nm to evaluate its influence on the device’s breakdown voltage.

Key physical models applied in the simulation included:

- Shockley–Read–Hall (SRH) recombination to model the carrier recombination processes;
- Mobility models accounting for temperature and electric field dependence;
- Bandgap-narrowing effects in the presence of high doping concentrations.

**Figure 1:**
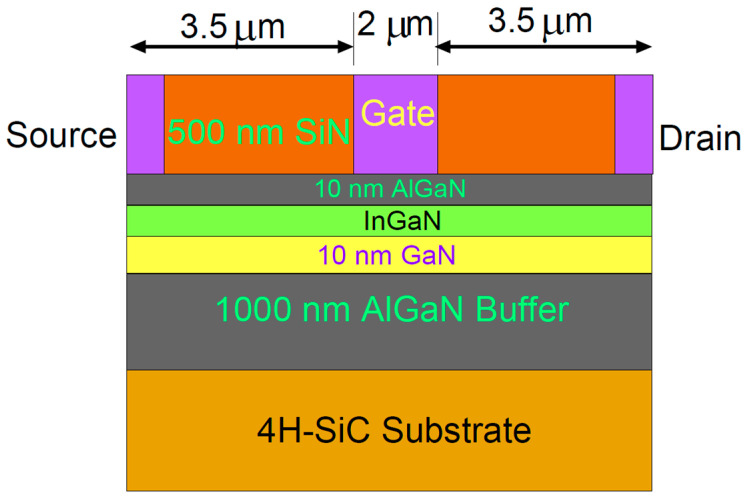
Structure of AlGaN/InGaN/GaN/AlGaN/SiC HEMT with InGaN/GaN coupling channel and AlGaN back-barrier layer.

The donor concentration was set to 1 × 10^18^ cm⁻^3^ near the source and drain regions to simulate the actual doping profile in the HEMT structure. The simulation employed a gate length of 2 µm, and the electrical characteristics, including transfer and output curves, were extracted from the simulated model. The primary focus of the simulation was on the breakdown voltage and how it responded to variations in the InGaN layer’s thickness and composition. Figure 1 illustrates the structure of the simulated device.

## 3. Results and Discussion

**Figure 2:**
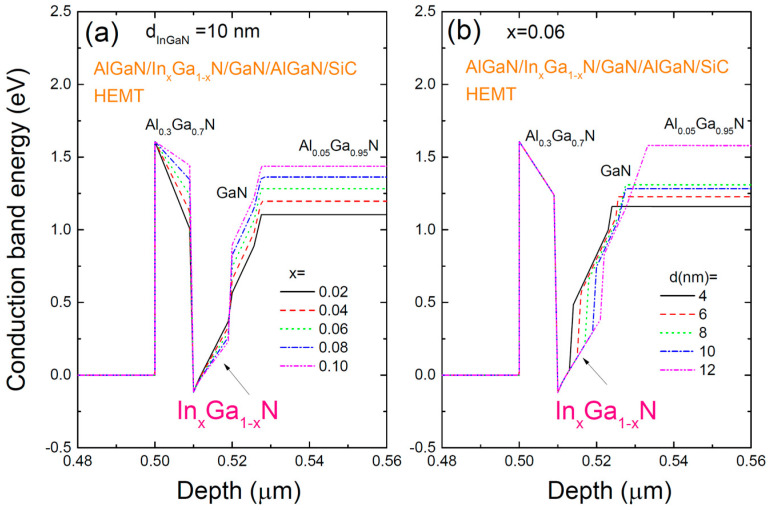
Conduction band profiles for the AlGaN/InGaN/GaN/AlGaN/SiC HEMT structure with (**a**) various In compositions and (**b**) different thicknesses of the InGaN layer. The conduction band profile is important in understanding the electron confinement within the well because the carrier distribution depends on the well depth and the well width.

Figure 2 shows the conduction band profile for the AlGaN/InGaN/GaN/AlGaN/SiC HEMT structure with respect to (a) various In compositions and (b) different InGaN layer thicknesses. The addition of InGaN, which has a smaller bandgap, results in deeper potential wells that take a U-shaped form, with the GaN layer acting as a natural back-barrier. As the In composition increases, the well depth deepens, which enhances electron confinement within the well. This increase in well depth is expected to increase the electron concentration within the well. Additionally, as the thickness of the InGaN layer increases, the width of the well expands, which could lead to a broader distribution of electrons. However, the impact of these changes on device characteristics would require further optimization. The 1 µm thick Al_0.05_Ga_0.95_N back-barrier buffer layer is also expected to improve carrier density within the channel while suppressing leakage currents, thus contributing to device stability.

**Figure 3:**
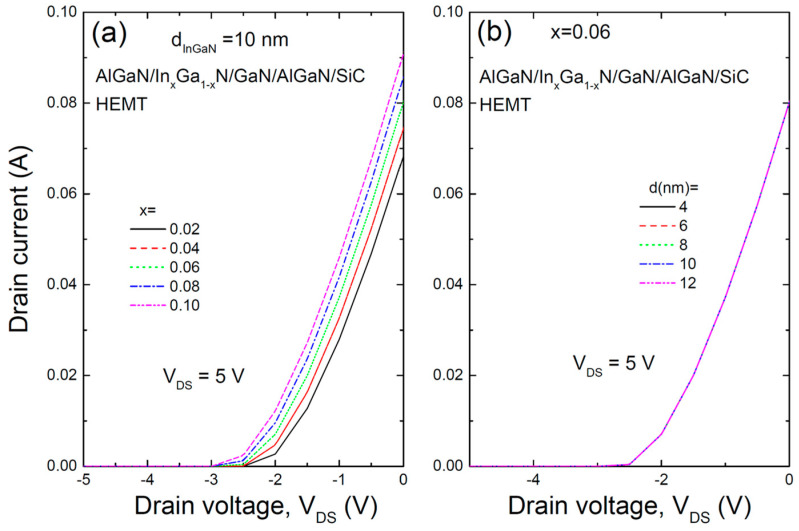
Transfer characteristics of the AlGaN/InGaN/GaN/AlGaN/SiC HEMT structure for (**a**) various In compositions and (**b**) different thicknesses of the InGaN layer.

Figure 3 illustrates the transfer characteristics of the AlGaN/InGaN/GaN/AlGaN/SiC HEMT structure for (a) various In compositions and (b) different InGaN thicknesses. The gate voltage (V_GS_) was varied from −5 V to 0 V with a drain-source voltage (V_DS_) of 5 V. The results show that as the In composition increases, the drain current rises for the same drain voltage, whereas the transfer characteristics are largely unaffected by the thickness of the InGaN layer. Non-linearity between the drain voltage and current causes distortion of the input signal, which leads to unnecessary harmonics or intermodulation distortion in the output signal. This distortion can degrade the quality of communication signals and negatively affect system performance. Maintaining linearity reduces signal distortion, resulting in a cleaner output signal. Improving the linearity between drain voltage and current in HEMTs can be achieved through various approaches, including device structure, material selection, and circuit design [12,13].

Low subthreshold swing (SS) and drain-induced barrier lowering (DIBL) are desirable in HEMTs. Although not shown in this figure, the SS, calculated from a log-scale plot, increases as the In composition increases from 230 mV/dec for x = 0.04 to 790 mV/dec for x = 0.1. However, the SS remains largely unaffected by the InGaN layer thickness. Similarly, DIBL is near zero, indicating a minimal threshold voltage shift with increasing drain voltage.

**Figure 4:**
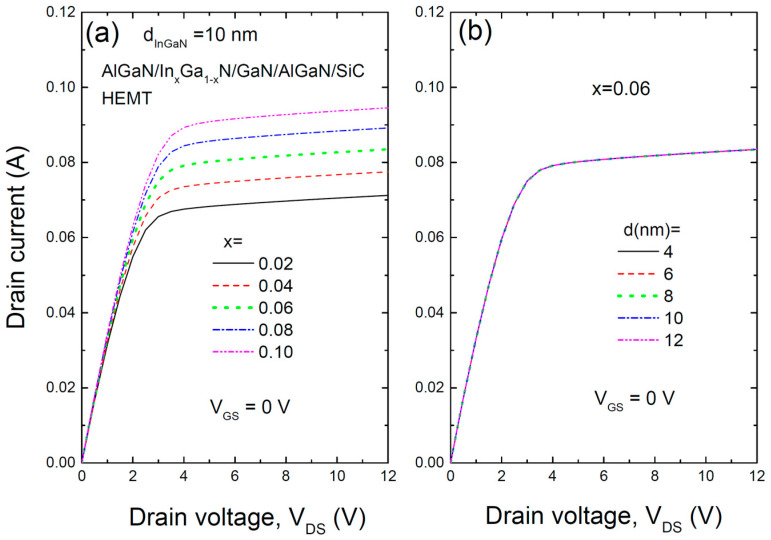
I-V output characteristics of the AlGaN/InGaN/GaN/AlGaN/SiC HEMT structure for (**a**) various In compositions and (**b**) different thicknesses of the InGaN layer, calculated at zero gate voltage.

Figure 4 presents the output characteristics (I-V) of the AlGaN/InGaN/GaN/AlGaN/SiC HEMT structure for (a) various In compositions and (b) different thicknesses of the InGaN layer, calculated at a gate voltage of 0 V. High-output current density (I_DS,max_) and high breakdown voltage (V_BR_) are critical for achieving high power density in RF power amplifiers. The results show that the drain current increases as the In composition increases, rising from 0.069 A for x = 0.02 to 0.093 A for x = 0.10. However, similar to the transfer characteristics, the output characteristics are largely unaffected by the thickness of the InGaN layer.

To optimize the threshold drain voltage for saturation in GaN HEMT devices, multiple promising approaches have been identified. One effective method is adjusting the gate recess depth, which allows for precise control over the threshold voltage. This adjustment results in a positive shift in the threshold voltage, effectively addressing challenges associated with normally on operation while enhancing gate control [14]. Another valuable technique is fluorine plasma treatment, which modulates the threshold voltage in AlGaN/GaN HEMTs by incorporating fluorine ions. This approach successfully adjusts the threshold voltage without adversely affecting device performance, particularly at elevated drain-source saturation currents [15]. In p-GaN gate HEMTs, the use of an oxidation interlayer has been shown to significantly increase both threshold voltage and gate breakdown reliability, making it highly applicable for high-power environments [16]. Furthermore, in GaN HEMTs with mixed conductive channels, adjusting the p-type doping concentration effectively optimizes the threshold voltage, balancing both the saturation current capability and the desired threshold levels [17]. These optimization strategies are crucial for high-power and RF applications, as precise threshold voltage tuning is instrumental in improving both device reliability and operational efficiency.

**Figure 5:**
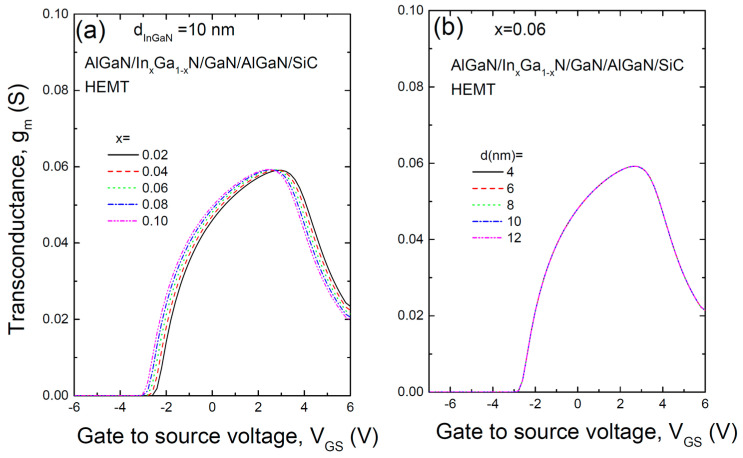
Transconductance (gm) of the AlGaN/InGaN/GaN/AlGaN/SiC HEMT structure for (**a**) various In compositions and (**b**) different thicknesses of the InGaN layer.

Figure 5 shows the transconductance (g_m_) for the AlGaN/InGaN/GaN/AlGaN/SiC HEMT structure as a function of (a) various In compositions and (b) different InGaN layer thicknesses. This transconductance, which represents the rate of change in drain current with respect to gate voltage (gm = ∂I_DS_/∂V_GS_), is essential for high-speed HEMT operation and enhancing gate efficiency [18]. The transconductance increases with gate voltage, then drops rapidly beyond a certain point, a phenomenon known as the transconductance roll-off, which is a significant issue in nanoscale HEMTs. Reasons for the drop are known to be a decreased saturation velocity of 2DEG with high gate voltage, non-linear source access resistance, and optical phonon emission at a high drain current [19,20]. A bell-shaped transconductance provides advantages such as maximizing gain, improving linearity, reducing signal distortion, and maintaining stability, playing a crucial role in optimizing transistor performance [21,22]. Managing gate length is critical in addressing transconductance roll-off in HEMTs. While short gate lengths are essential for high-frequency and high-speed operation, they introduce challenges like velocity saturation, short-channel effects, and electric field concentration [23,24,25,26]. Strategies such as gate length optimization, field plates, recessed gates, and high-mobility channel materials can significantly mitigate transconductance roll-off and enhance overall device performance. The gate voltage swing (GVS), which represents the gate bias range where the transconductance reaches 80% of its maximum value, is calculated to be around 4.1 V. A high GVS is crucial for linear RF applications. While this study does not address gate length variation, which generally results in flatter transconductance, the peak transconductance observed was 0.059 S for a gate length of 2 µm. The transconductance, like the transfer characteristics, was largely unaffected by the thickness of the InGaN layer.

**Figure 6:**
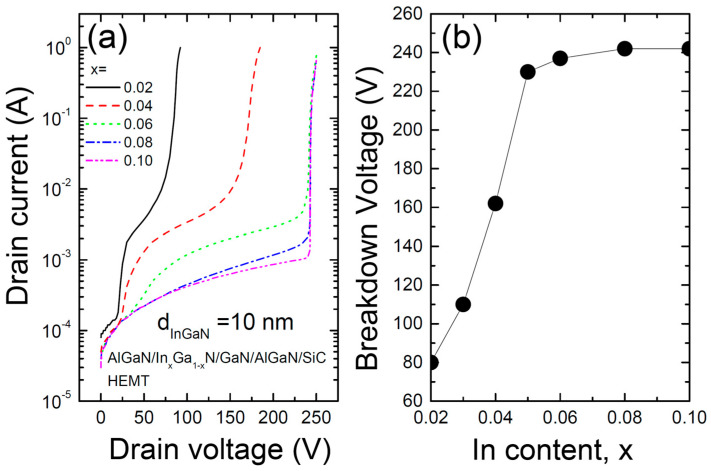
(**a**) Relationship between drain voltage and current and (**b**) variation in breakdown voltage (V_BR_) as a function of In content for the AlGaN/InGaN/GaN/AlGaN/SiC HEMT structure with various In compositions in the InGaN layer.

Figure 6 shows (a) the relationship between the drain voltage and current for various In compositions in the InGaN layer of the AlGaN/InGaN/GaN/AlGaN/SiC HEMT structure and (b) the variation in the breakdown voltage (VBR) as a function of the In composition. Selberherr’s impact ionization model was used in the simulation of the HEMT [27,28,29,30,31]. The breakdown voltage is shown to be highly dependent on the In composition. Specifically, as the In composition increases, the breakdown voltage rapidly increases. This can be explained by the fact that as the In composition increases, the bandgap decreases, and at the same time, the internal field due to spontaneous and piezoelectric polarization increases, deepening the potential well. As a result, carrier confinement increases while the leakage current into the buffer is suppressed. However, when the In composition increases beyond a certain point, this effect saturates, and the breakdown voltage also shows saturation. Therefore, it has been found that if the In composition is set to 0.06 or higher, a high breakdown voltage can be achieved. As the In content increases, the bandgap of InGaN decreases. This reduction in the bandgap causes an electric field dispersion effect. As a result, the device can withstand higher voltages before reaching breakdown. However, with increasing the In content, the electron density within the channel can also rise. An increase in electron density enhances the channel’s responsiveness to the electric field, which can intensify electric field concentration. Consequently, at a certain level of In content, the charge density in the channel becomes too high, which may hinder further increases in breakdown voltage, leading to voltage saturation [32,33,34].

**Figure 7:**
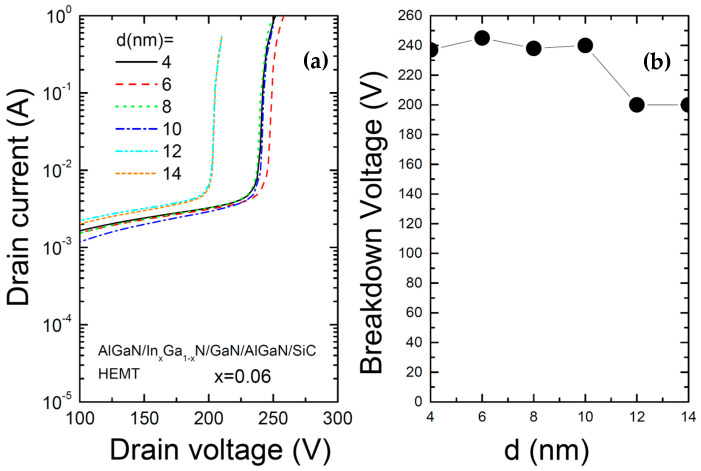
(**a**) Relationship between drain voltage and current and (**b**) variation in breakdown voltage (V_BR_) as a function of InGaN layer thickness for the AlGaN/InGaN/GaN/AlGaN/SiC HEMT structure.

Figure 7 shows (a) the relationship between the drain voltage and current for various thicknesses of the InGaN layer in the AlGaN/InGaN/GaN/AlGaN/SiC HEMT structure and (b) the variation in the breakdown voltage (V_BR_) as a function of In composition. It is shown that up to a certain increase in thickness, the breakdown voltage remains almost constant. However, when the thickness exceeds 10 nm, the breakdown voltage decreases. The variation in the breakdown voltage with thickness is relatively small compared to the variation with In composition. The reason the breakdown voltage decreases for larger thicknesses is believed to be that the wider potential well, due to the increased thickness, reduces carrier confinement. Therefore, it is desirable to keep the thickness below 10 nm.

Since power devices usually operate in high-temperature environments, temperature impacts the breakdown voltage and performance of the AlGaN/InGaN/GaN/AlGaN/SiC HEMT structure. The effect of temperature on the breakdown voltage of HEMTs is primarily related to the decrease in electron mobility, reduction in bandgap, increase in recombination, and changes in the electric field distribution. These factors can degrade the overall performance of HEMTs, especially diminishing stability and reliability in high-temperature environments [35,36,37,38,39]. Several papers showed that the results using the Silvaco TCAD program are in good agreement with the experimental results. These papers demonstrate that Silvaco TCAD simulations for GaN HEMTs closely match experimental data across various performance metrics, validating the program’s effectiveness in device modeling and reliability assessments [1,40,41,42,43,44].

## 4. Conclusions

In conclusion, the effects of In content and InGaN layer thickness on the performance of AlGaN/InGaN/GaN/AlGaN/SiC HEMTs were investigated through simulation. The results demonstrate that the breakdown voltage is strongly influenced by the In content in the InGaN layer, with the breakdown voltage increasing as the In content increases. This trend is attributed to the enhanced electron confinement and deeper potential wells, driven by the increased polarization fields associated with higher In compositions. However, the breakdown voltage saturates at an In content of 0.06, suggesting that further increases in In composition provide diminishing returns in terms of performance. Additionally, it was found that while increasing the thickness of the InGaN layer has minimal impact on the device’s electrical characteristics up to a point, exceeding a thickness of 10 nm results in a noticeable reduction in breakdown voltage, likely due to reduced carrier confinement.

## Data Availability

The datasets used and/or analyzed during the current study are available from the corresponding author on reasonable request.
